# Identification and Validation of Prognostic Factors of Lipid Metabolism in Obstructive Sleep Apnea

**DOI:** 10.3389/fgene.2021.747576

**Published:** 2021-11-22

**Authors:** Lu Peng, Xiaodi Wang, Dan Bing

**Affiliations:** ^1^ Department of Otorhinolaryngology Head and Neck Surgery, Tongji Hospital Affiliated to Tongji Medical College of Huazhong University of Science and Technology, Wuhan, China; ^2^ Department of Otorhinolaryngology Head and Neck Surgery, Second Affiliated Hospital of Guangxi Medical University, Nanning, China

**Keywords:** lipid metabolism, obstructive sleep apnea, immunologic factors, macrophage activation, microarray analysis

## Abstract

**Background:** Obstructive sleep apnea (OSA) is considered to be an independent factor affecting lipid metabolism. This study explored the relationship between immune genes and lipid metabolism in OSA.

**Methods:** Immune-related Differentially Expressed Genes (DEGs) were identified by analyzing microarray data sets from the Gene Expression Omnibus (GEO) database. Subsequently, we conducted protein-protein interaction (PPI) network analysis and calculated their Gene Ontology (GO) semantic similarity. The GO, Kyoto Encyclopedia of Genes and Genomes (KEGG) pathways, Disease Ontology (DO), gene set enrichment analysis (GSEA), and gene set variation analysis (GSVA) were employed for functional enrichment analyses and to determine the most significant functional terms. Combined with the results of boruta and random forest, we selected predictors to build a prognostic model, along with seeking out the potential TFs and target drugs for the predictive genes.

**Results:** Immune-related DEGs included 64 genes upregulated and 98 genes downregulated. The enrichment analysis might closely associate with cell adhesion and T cell-mediated immunity pathways and there were many DEGs involved in lipid and atherosclerosis signaling pathways. The highest-ranking hub gene in PPI network have been reported lowly expressed in OSA. In line with the enrichment analysis, DO analysis reveal that respiratory diseases may be associated with OSA besides immune system disorders. Consistent with the result of the KEGG pathway, the analysis of GSVA revealed that the pro-inflammation pathways are associated with OSA. Monocytes and CD8 T cells were the predominant immune cells in adipose tissue. We built a prognostic model with the top six genes, and the prognostic genes were involved in the polarization of macrophage and differentiation of T lymphocyte subsets. In vivo experimental verification revealed that EPGN, LGR5, NCK1 and VIP were significantly down-regulated while PGRMC2 was significantly up-regulated in mouse model of OSA.

**Conclusions:** Our study demonstrated strong associations between immune genes and the development of dyslipidemia in OSA. This work promoted the molecular mechanisms and potential targets for the regulation of lipid metabolism in OSA.

## Introduction

Obstructive Sleep Apnea (OSA) is a common disease characterized by repeated episodes of upper airway closure during sleep. The symptoms of OSA, such as snoring, nocturnal awakening, nocturia, and daytime sleepiness has low specificity to distinguish (Patel, 2019). The apnea-hypopnea index (AHI) and hypopnea per hour of sleep is the key metric to measure OSA (Jordan et al., 2014). Overnight polysomnography is the best test of OSA, as the procedure is dedicated and expensive. Atypical symptoms and inconvenient detection methods lead to a low diagnostic rate of OSA. OSA affects 9–38% of the adult population, from 6 to 19% in women and 13–33% in men (Senaratna et al., 2017).

OSA is associated with increased risk for hypertension, coronary artery disease, heart failure, stroke, type 2 diabetes, and fatty liver diseases (Tan et al., 2018; Chung et al., 2021; Yeghiazarians et al., 2021). Nowadays, more and more evidence shows that in the treatment of those diseases, OSA should be paid more attention to (Tan et al., 2018; Yeghiazarians et al., 2021). There has been a great interest in the interaction between OSA and metabolic dysfunction. Patients with OSA usually have abnormal metabolism of glucose and lipids. Though obesity is one of the main risk factors of OSA, many investigations have shown that OSA can have an independent effect on dyslipidemia as well as obesity (Karkinski et al., 2017; Silva et al., 2018; Alterki et al., 2020). In non-obese patients, OSA could aggravate abnormal lipid metabolism (Karkinski et al., 2017). Dysregulation lipid profiles are related to sleep hypoxemia even in mild OSA (Silva et al., 2018). But in obese patients, the role of OSA in the changes of dyslipidemia is not as important as in non-obese patients (Karkinski et al., 2017). However, it has also been reported that after eliminating interference factors, only severe OSA had an independent association with dyslipidemia (Martínez-Cerón et al., 2021). Treatment with OSA, either multilevel sleep surgery or continuous positive airway pressure (CPAP) therapy, has a positive impact on the metabolic status (Alterki et al., 2020; Simon et al., 2020). So, the question is, which key factors through what signaling pathways contribute to abnormal lipid metabolism in patients with OSA.

Lots of studies using high throughput microarray to analyze the differential expression genes and functional pathways related to the mechanisms and consequences of OSA. A previous study took two systems biology approaches to detect hub proteins associated with OSA in subcutaneous and visceral fat tissues. The hub genes were different between using biased methods and unbiased methods, because of the nature of the two approaches (Liu et al., 2011). Two studies were obtained the same result that the olfactory transduction pathway plays an important role in OSA using visceral adipose tissues (Gu et al., 2019) and subcutaneous adipose tissues (Cao et al., 2021) respectively. Yet, the different biomarkers and enriched pathways were using the same microarray data from visceral adipose tissues (GSE38792) (Chen et al., 2015; Gu et al., 2019), for the small sample size increased the false-positive of the results. Moreover, although a series of bioinformatics analyses has thoroughly investigated the potential biomarkers and functional pathways of OSA in adipose tissues, they remain to use cross analysis in various datasets to explore the possible mechanisms. There is a high false-positive rate using a single dataset or single method that may contribute to discordant results across these studies. Previous studies have shown that inflammatory is involved in the development of OSA (Gharib et al., 2020; Liu et al., 2020), the landscape of immune infiltration in OSA has not been entirely revealed. Accordingly, we conducted cross-analysis in immune-related biomarkers and the predicted target drugs of treatment in OSA dyslipidemia.

In the present study, we discover the key immune molecules and signaling pathways involved in lipid metabolism and identify immune molecule-related transcription factors (TFs) and drug targets. To obtain more accurate results, we downloaded two microarray datasets from the Gene Expression Omnibus (GEO) database, and then analyzed and verified them. We obtained immune-related differentially expressed genes (DEGs) between normal and OSA groups. With these DEGs, we conducted protein-protein interaction (PPI) network analysis and calculated their Gene Ontology (GO) semantic similarity. Gene Ontology (GO), Kyoto Encyclopedia of Genes and Genomes (KEGG) pathways, Disease Ontology (DO), gene set enrichment analysis (GSEA), and gene set variation analysis (GSVA) were employed for functional enrichment analyses and to determine the most significant functional terms. Combined with the results of boruta and random forest, we selected predictors to build a prognostic model, along with seeking out the potential TFs and target drugs for the predictive genes. The aim of this study was to provide a theoretical basis for immune genes that affected lipid metabolism in OSA.

## Material and Methods

### Data Collection and Processing

The datasets were obtained from the Gene Expression Omnibus database (GEO) (http://www.ncbi.nlm.nih.gov/geo/). GSE135917 (Gharib et al., 2020) and GSE38792 (Gharib et al., 2013) microarray datasets were performed on the same platform GPL6244 (HuGene-1_0-st; Affymetrix Human Gene 1.0 ST). GSE135917 contained fifty subcutaneous adipose tissue samples including normal controls (n = 8) and OSA patients without treatment (n = 34). GSE38792 contained eighteen visceral adipose tissue samples including normal controls (n = 8) and OSA patients without treatment (n = 10). We used GSE135917 as the training set and GSE38792 as the testing set. The clinical and demographic characteristics of the study patients in GSE135917 was shown in Table 1.

**TABLE 1:** Clinical and demographic characteristics of patients in two datasets.

	GSE135917			GSE38792	
	Group1 (individuals undergoing ventral hernia repair surgery)		Group2 (individuals diagnosed with OSA getting initiated on CPAP therapy)	Control (n = 8)	OSA (n = 10)
	Control (n = 8)	OSA (n = 10)	OSA (n = 24)		
Age (years)	54.5 ± 11.6	56.1 ± 10.8	49.4 ± 10.9	54.5 ± 11.6	56.1 ± 10.8
Gender					
Male	1	3	13	1	3
Female	7	7	11	7	7
Body mass index (kg/m^2^)	35.2 ± 5.8	36.1 ± 9.3	42.6 ± 9.4	35.2 ± 5.8	36.1 ± 9.3
RDI or AHI (events/hour)	0.6 ± 0.5	19.2 ± 25.9	41.5 ± 23.8	0.6 ± 0.5	19.2 ± 25.9
Diabetes	0	3	5	0	3
Hypertension	3	3	9	3	3
Heart disease	0	0	2	0	0

Raw data were downloaded using the GEOquery package (Davis and Meltzer, 2007) and analyzed using the oligo package (Carvalho and Irizarry, 2010) of Bioconductor in R version 4.1.0. The data were normalized with the RMA method and probe IDs were converted into gene names according to the platform annotation information.

### Differentially Expressed Genes Analysis

Limma package (Ritchie et al., 2015) in R was used to identify DEGs between OSA and normal adipose tissues. Genes with adjusted *p*-value < 0.01 were considered to be statistically significant.

The immune-related gene list was obtained from the ImmPort database (http://www.immport.org) (Bhattacharya et al., 2014). Then the gene set with immune-related genes was identified in the GSE135917 and the immune-related genes with adjusted *p*-value < 0.01 were considered to be statistically significant.

### Protein-Protein Interaction Network Construction With Immune-Related Differentially Expressed Genes

The STRING database (https://string-db.org/) (von Mering et al., 2003) is a biological database and web resource of known and predicted protein-protein interactions. We uploaded the immune-related DEGs to the STRING database. The species was set as Homo sapiens and the minimum interaction score was 0.4 to build a protein interaction network. The PPI network of the immune-related DEGs was visualized with Cytoscape 3.8.2 software (Shannon et al., 2003).

### Calculation of Gene Ontology Semantic Similarity

GO terms include biological process (BP), molecular function (MF), and cellular component (CC). The GO semantic similarity score can be applied to quantify the functional similarity between genes. To assess immune-related gene functional similarity, we calculated semantic similarity scores of GO terms using the R GOSemSim package (Yu et al., 2010; Yu, 2020). MgeneSim automatically removes genes without annotations and computed the semantic similarity among GO terms. The functional similarity score of the target gene is calculated as follows:

$$\;\mathrm{Fsim}=\sqrt[{3}]{simbp\;*\;simcc\;*\;simmf}$$

### Gene Ontology and Pathway Analysis

To reveal the functions and pathways of immune-related DEGs, GO and KEGG pathway analyses were performed using the R clusterProfiler package (Yu et al., 2012). Significant KEGG pathways and participating genes were visualized with the R pathview package (Luo and Brouwer, 2013). In all enrichment analyses, Benjamini-Hochberg (BH) adjustment to calculate the false discovery rate (FDR) was applied. A q-value < 0.05 was set as the cutoff criterion.

DOSE (Yu et al., 2015) is an R package for disease ontology semantic and enrichment analysis. We used the DOSE R package to analyze the enrichment of immune-related DEGs with Disease Ontology (DO) terms.

### Gene Set Enrichment Analysis and Gene Set Variation Analysis

Gene set enrichment analysis was performed using the R clusterProfiler package (Yu et al., 2012). *p* values were adjusted by the BH method. We used FDR (false discovery rate) < 0.1, and *p*-value < 0.01 as the threshold to determine significant enrichment of the gene sets. Then Gene Set Variation Analysis (GSVA) (Hänzelmann et al., 2013), a nonparametric unsupervised method, was used to display differential enrichment pathways between normal controls and OSA patients. A *p*-value < 0.01 was set as the cutoff criterion. In this study, we used the R package “GSVA” to explore KEGG pathways of immune-related genes. Gene terms were considered statistically significant, and a heatmap was generated using R.

### Prognostic Model Building and Validation

Boruta feature selection (“Boruta” package in R) (Miron B. Kursa and Rudnicki, 2010) was used on the training dataset (GSE135917) to identify the immune-related genes that contribute significantly to OSA. Random forest was implemented in R using the randomForest package (Liaw and Wiener, 2001), and important immune-related genes were selected as features that could construct a prognostic model.

Based on the results of boruta and random forest, the selected set of predictors were used to construct a prognostic model. The receiver operating characteristic (ROC) curve was used to confirm the performance of the model and the area under the curve (AUC) was estimated in the training dataset (GSE135917), and the GSE38792 dataset was applied to verify the established prognostic model. At last, we analyzed the expression of predictors between normal and OSA groups in the two datasets.

### Immune Cell Infiltrate Analysis

To understand the immune microenvironment of adipose tissue, we analyzed the differential expression of different types of immune cells. The CIBERSORTx algorithm (Newman et al., 2015) was used to calculate and analyze the immune microenvironment of adipose tissue involved in OSA and normal controls.

### Correlation Analysis Between Predictors and Immune Cells

To further understand the relationship between predictors and immune cells, Pearson’s correlation was performed to analyze the correlation between the expression value of predictors and the different types of immune cells.

### Transcription Factors and Target Drugs Analysis

To further investigate the transcription factor binding motifs of predictors, the iRegulon (Janky et al., 2014) software was used. The set of predictors was submitted to iRegulon and analyzed using the following options: minimum NEScore = 5.0. The results were visualized using Cytoscape software.

Furthermore, we identified drug-gene interactions using Drug-Gene Interaction Database (DGIdb) (Cotto et al., 2018). The list of predictors was uploaded to DGIdb and matched with drugs that could be the potential therapeutic targets of OSA.

### Animal Model and Chronic Intermittent Hypoxia Protocols

C57BL/6J adult male mice (8 weeks old) were purchased from the Model Animal Research Center of Tongji Medical College of Huazhong University of Science and Technology (Wuhan, China). Animals were randomly assigned to control and OSA groups (n = 6 animals/group). The OSA group mice were exposed to 4 weeks of CIH (8 daylight hours per day, 10:00 am to 6:00 pm), whereas the control group was maintained under normal oxygenation conditions. The animal study was approved by the Institutional Ethics Committee for Animal Research of Tongji Medical College, Huazhong University of Science and Technology. All procedures conformed to the Guide for the Care and Use of Laboratory Animals.

Gas-control delivery equipment was installed to regulate nitrogen and oxygen flow into the customized chamber. The equipment was composed of sensors for O2 and gas injectors. During each 510-s cycle there included 4 stages. In stage 1, with N2 infused into the chamber, the concentration of O2 lowered from 21 to 5% in 150 s, and then maintained at 5% for 120 s in stage 2. In stage 3, the chamber was infused with O2 for 120 s to restore O2 to an ambient concentration of 21%, and it was maintained in stage 4 until the beginning of the next CIH cycle.

### Reverse Transcription-Polymerase Chain Reaction

Visceral adipose tissue was collected and total mRNA and was extracted using TRIzol Reagent following the manufacturer’s protocol. The extracted mRNA (1 μg) was reverse transcribed into cDNA using. Real-time PCR was performed on a LightCycler System 2.0 (Roche, Mannheim, Germany) using SYBR Premix EX Taq kit (Takara, Dalian, China). RT-PCR was performed at 95°C for 5 min, then 95°C (45 s), 56°C (30 s), and 72°C (45 s) followed by a 10 min extension at 72°C for 40 cycles. Each sample was run in triplicate and averaged. The relative gene expression was calculated by the 2-△△Ct method.

The primer sequence is as follows: EPGN forward primer: 5′-GGG​GGT​TCT​GAT​AGC​AGT​CTG-3′, reverse primer: 5′-TCG​GTG​TTG​TTA​AAT​GTC​CAG​TT-3’. LGR5 forward primer: 5′- CCT​ACT​CGA​AGA​CTT​ACC​CAG​T-3′, reverse primer: 5′- GCA​TTG​GGG​TGA​ATG​ATA​GCA-3’. NCK1 forward primer: 5′- TCC​TGC​TGA​TGA​TAG​CTT​TGT​TG-3′, reverse primer: 5′- ACG​ATC​ACC​TTG​GTC​CCT​TTT​AT-3’. PGRMC2 forward primer: 5′- TGG​GAA​AGT​CTT​CGA​CGT​GAC-3′, reverse primer: 5′- GTG​CAT​CCT​TAT​CCA​GGC​AGA-3’. VIP forward primer: 5′- AGT​GTG​CTG​TTC​TCT​CAG​TCG-3′, reverse primer: 5′- GCC​ATT​TTC​TGC​TAA​GGG​ATT​CT-3’. β-actin forward primer: 5′- GCG​CAA​GTA​CTC​TGT​GTG​GA-3′, reverse primer: 5′-GAAAGGGTGTAAAAC GCAGC-3’.

## Results

### Data Pre-processing

**FIGURE 1:**
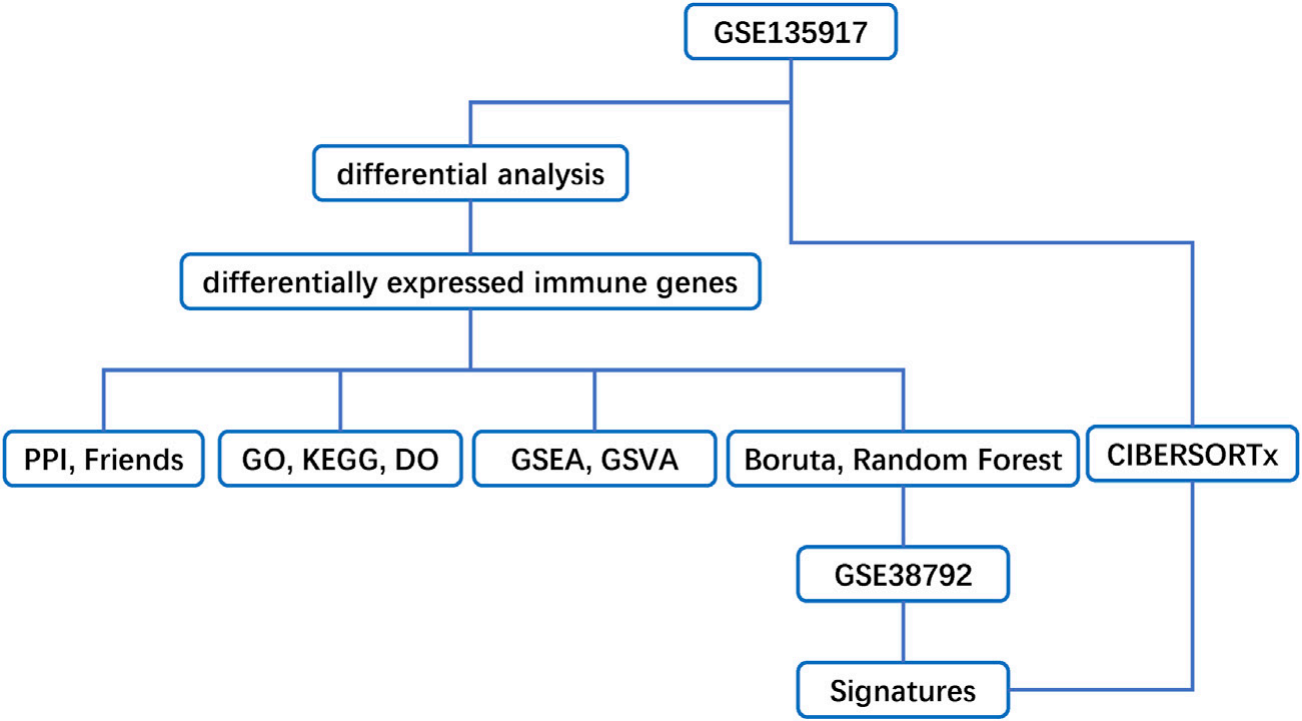
Flow chart of methodologies applied in the current study.

**FIGURE 2:**
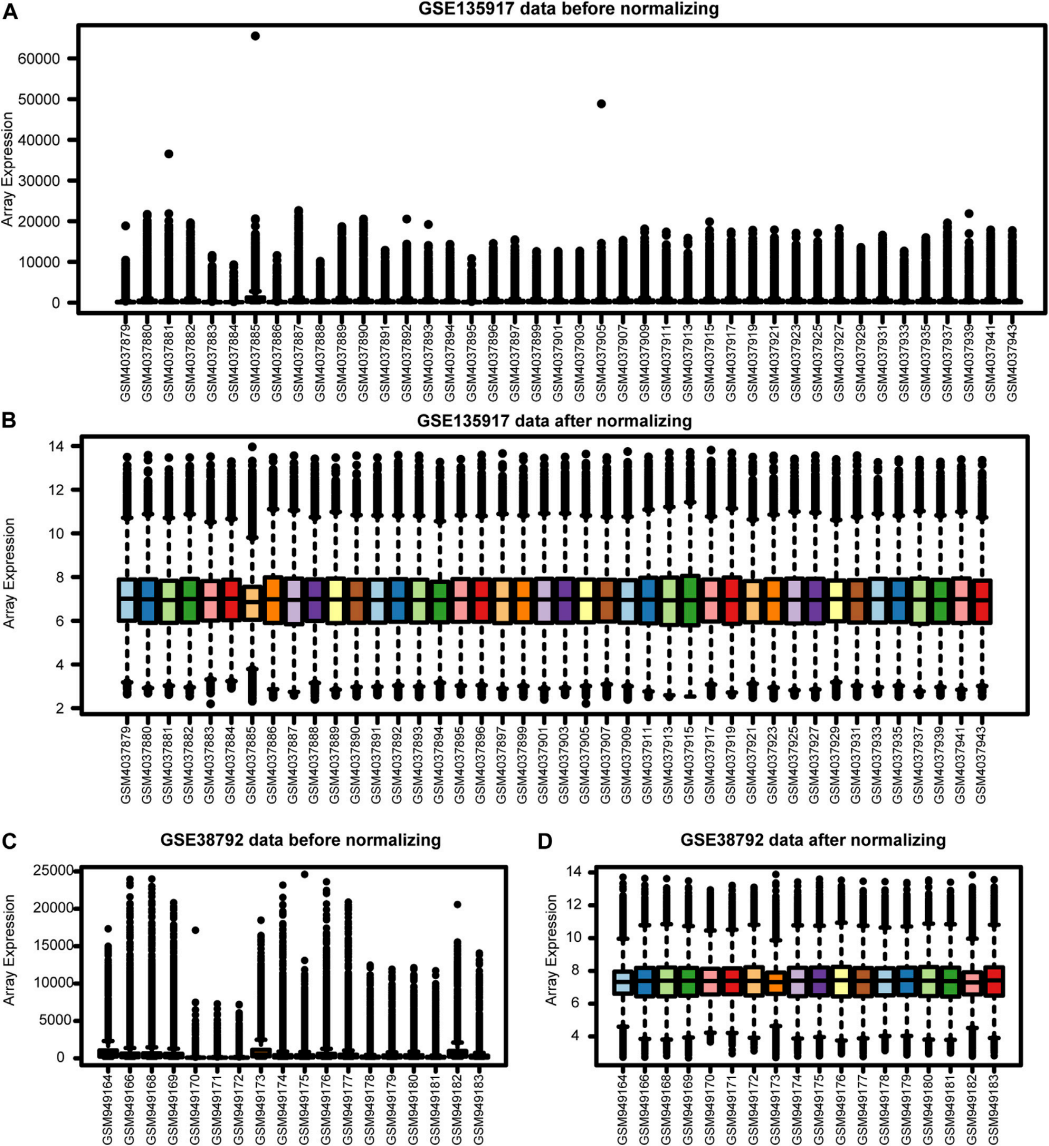
The Expression profiles before and after normalization. **(A)** GSE135917 data before normalization. **(B)** GSE135917 data after normalization. **(C)** GSE38792 data before normalization. **(D)** GSE38792 data after normalization.

Our workflows are shown in Figure 1. To eliminate batch expression difference, each data was normalized by the RMA method in R (Figure 2). The original expression values varied significantly between the samples and the mean values of gene expression for each sample were fundamentally the same after normalization.

### Identification of Differentially Expressed Genes in Obstructive Sleep Apnea

**FIGURE 3:**
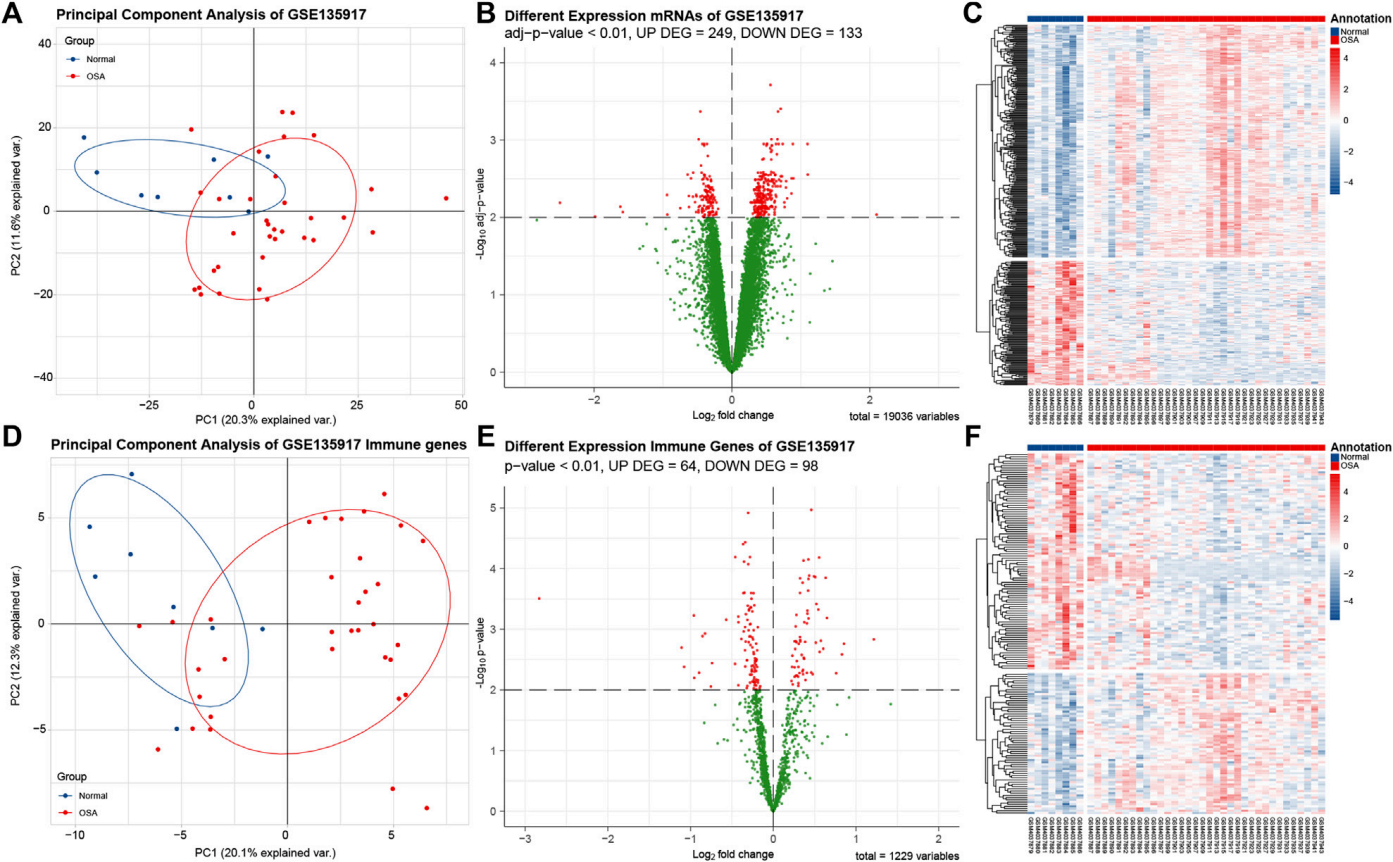
Analysis of DEGs and immune-related DEGs in data set GSE135917. **(A)** Principle component analysis (PCA) plot of 382 DEGs shows that samples be divided into two clusters. Blue dots indicate normal samples, red dots indicates obstructive sleep apnea (OSA) samples. **(B)** The volcano plot of differentially expressed genes (DEGs). Red dots represent significant different expression genes, and green dots represent no significant different expression genes. **(C)** The heatmap of DEGs. Each row represents one gene, and each column represents one sample. Red indicates that the expression of genes is relatively upregulated, and blue indicates that the expression of genes is relatively downregulated. **(D)**. PCA plot of 162 immune-related DEGs. **(E)** The volcano plot of immune-related DEGs. **(F)** The heatmap of immune-related DEGs.

The GSE135917 after normalization was utilized to obtain the differentially expressed genes between OSA and normal adipose tissues. With adj *p*-value < 0.01 as the screening threshold, DEGs were obtained with 249 genes upregulated and 133 genes downregulated. The volcano plot and heatmap of the DEGs are shown in Figures 3B,C. With adj *p*-value < 0.01 as the screening threshold, immune-related DEGs were obtained with 64 genes upregulated and 98 genes downregulated. The volcano plot and heatmap of the DEGs are shown in Figures 3E,F. In vocano plot, red dots represent significant different expression genes, and green dots represent no significant different expression genes. In heatmap, each row represents one gene, and each column represents one sample. Red indicates that the expression of genes is relatively upregulated, and blue indicates that the expression of genes is relatively downregulated.

We applied principle component analysis (PCA) on the DEGs (Figure 3A) and immune-related DEGs (Figure 3D). Unsupervised clustering of the two set DEGs showed that samples from normal and OSA could be divided into two main clusters. The result revealed that the features of OSA adipose tissue could be explained only by immune-related DEGs, then we used 162 immune-related DEGs to perform subsequent analysis.

### Protein-Protein Interaction Network Construction and Semantic Similarity Analysis

**FIGURE 4:**
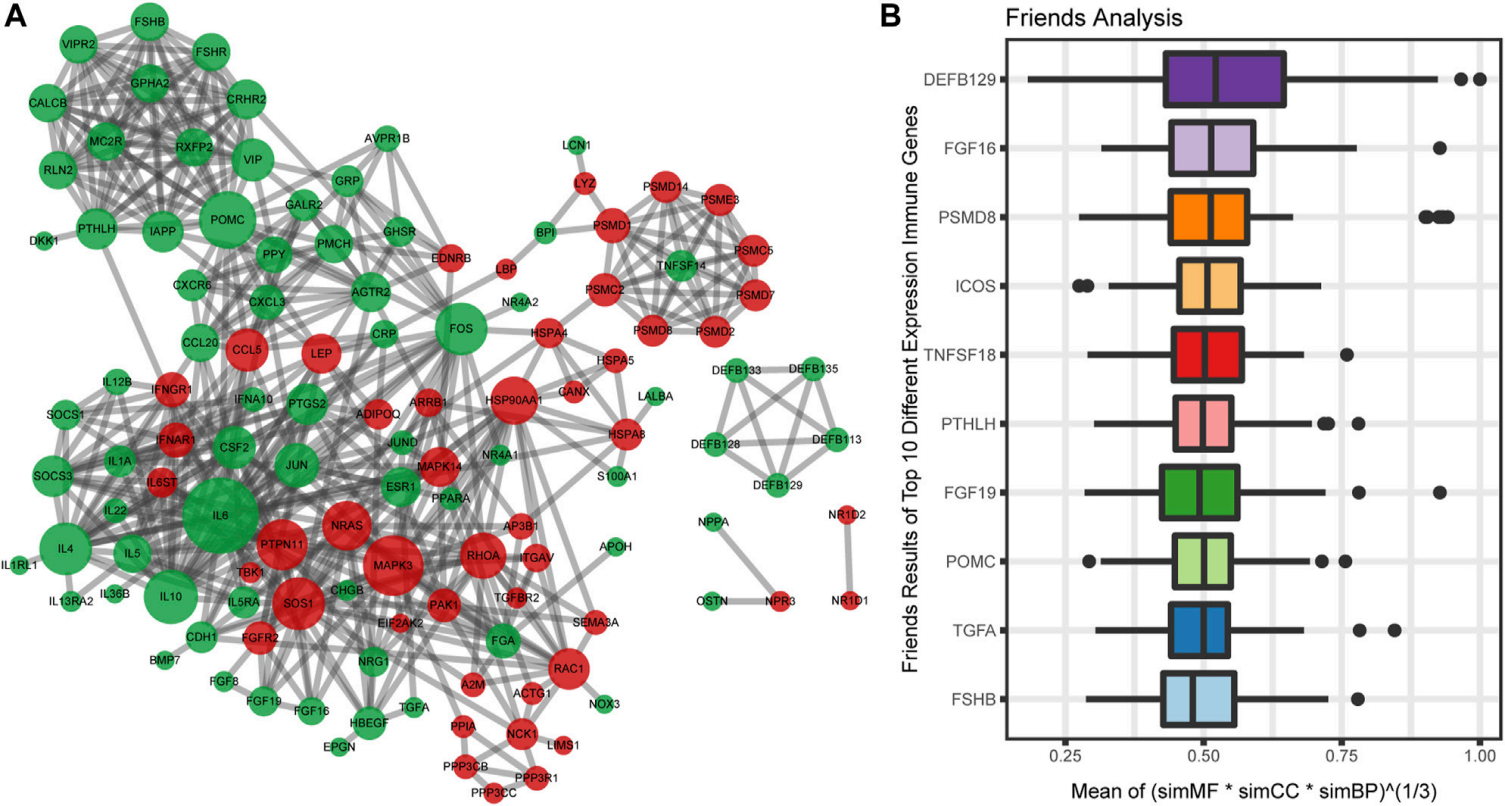
Protein-protein interactions (PPI) network construction and semantic similarity analysis of immune-related DEGs. **(A)** The PPI network of immune-related DEGs. Each circle represents a gene. The upregulated genes (red) and downregulated genes (green) are represented by circles. Different sizes indicate the core degree of genes in the PPI network, whereas bigger size indicates more important in the network. **(B)** Summary of functional similarities of the top 10 immune-related DEGs. The aggregate score is between 0 and 1. The higher the score is, the more similarity genes are.

To analyze the interaction among 162 immune-related DEGs, the STRING database was used. A total of 122 nodes and 496 edges were obtained with a combined score >0.7, as shown in Figure 4A. The top3 hub genes with the highest ranking were found: interleukin 6 (*IL6*), proopiomelanocortin (*POMC*), mitogen-activated protein kinase 3 (*MAPK3*).

We also calculated the average semantic similarity for immune-related DEGs in GO terms, including biological process (BP), molecular function (MF), and cellular component (CC) categories. Based on the average functional similarity, we ranked the top 10 genes among the immune genes (Figure 4B). Defensin beta 129 (*DEFB129*), fibroblast growth factor 16 (*FGF16*), and proteasome 26S subunit, non-ATPase 8 (*PSMD8*) were the top three genes potentially playing key roles in OSA.

### Gene Ontology, Kyoto Encyclopedia of Genes and Genomes Pathway, and Disease Ontology Terms Enrichment Analysis

**FIGURE 5:**
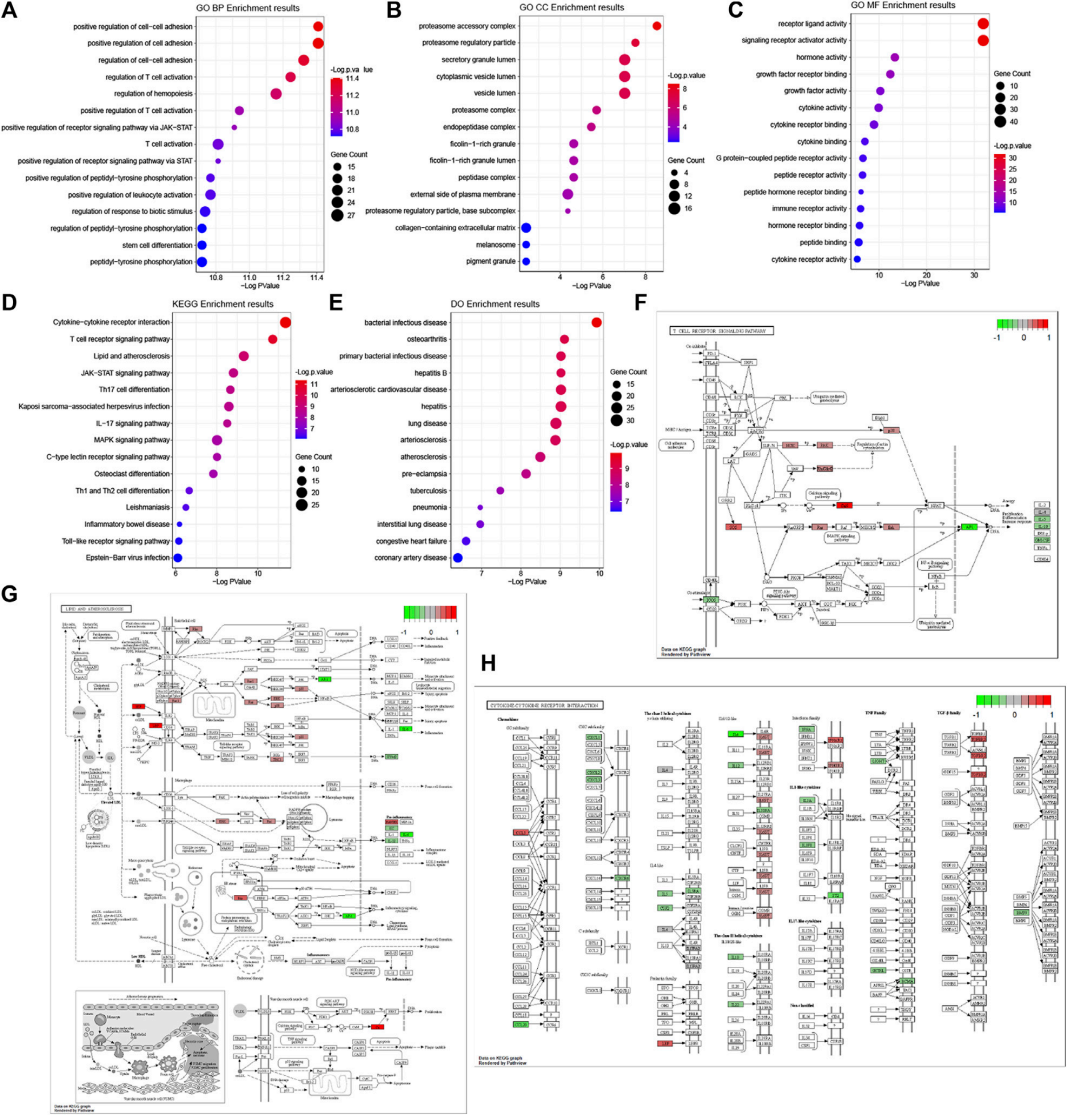
GO, KEGG pathway and Do terms enrichment analysis of immune-related DEGs. GO terms enrichment analysis of the DEGs, including BP **(A)**, CC **(B)**, and MF **(C)** categories. **(D)** KEGG pathway enrichment analysis of the DEGs. **(E)** Do terms enrichment analysis of DEGs. The size of the symbol represents the gene counts enriched in the signaling pathway. The color indicates the degree of significance. Signaling pathways of the T cell receptor signaling pathway **(F)**, lipid and atherosclerosis **(G)**, and cytokine-cytokine receptor interaction **(H)**. The genes significantly up-regulated filled in red color and down-regulated filled in green color.

The results of GO functional, KEGG pathway and Do terms enrichment analysis are shown in Figure 5. In the BP category of the GO enrichment analysis, immune-related DEGs were mainly enriched in items such as “positive regulation of cell-cell adhesion”, “positive regulation of cell adhesion”, and “regulation of cell-cell adhesion” (Figure 5A). In the CC category of the GO enrichment analysis, these genes were mainly enriched in items such as “proteasome accessory complex”, “proteasome regulatory particle”, and “secretory granule lumen” (Figure 5B). In the MF category of the GO enrichment analysis, these genes were mainly enriched in items such as “receptor ligand activity”, “signaling receptor activator activity”, and “hormone activity” (Figure 5C). Meanwhile, based on the results of the KEGG pathway enrichment analysis (Figure 5D), most of the immune-related DEGs were significantly enriched for the terms: “cytokine-cytokine receptor interaction” (Figure 5H), “T cell receptor signaling pathway” (Figure 5F), and “lipid and atherosclerosis” (Figure 5G). Therefore, the GO terms from BP and KEGG signaling pathways might closely associate with cell adhesion and T cell-mediated immunity pathways. Interestingly, among the differentially expressed genes, there were many involved in lipid and atherosclerosis signaling pathways.

We found DO terms mainly enriched in “bacterial infectious disease”, “osteoarthritis”, and “primary bacterial infectious disease” (Figure 5E). In line with the above GO-term and KEGG pathway analysis, disease ontology (DO) revealed that respiratory diseases may be associated with OSA besides immune system disorders.

### Pathway Enrichment Analysis of the Immune-Related Differentially Expressed Genes

**FIGURE 6:**
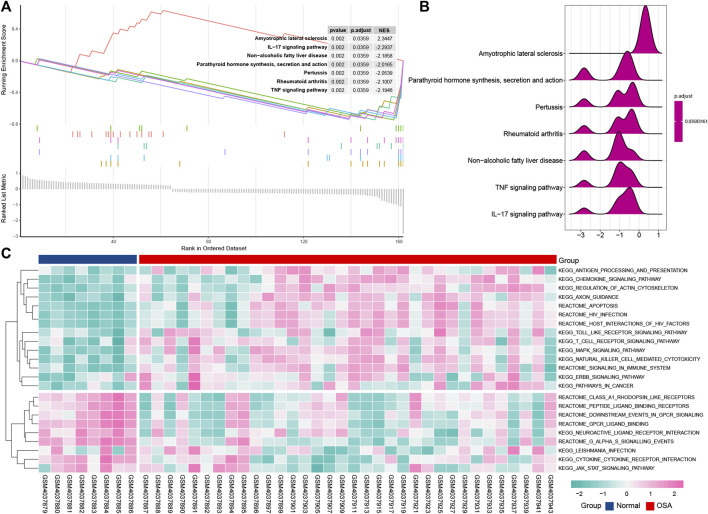
Pathway enrichment analysis of the immune-related DEGs. Gene set enrichment analysis (GSEA) displays the top 7 enriched pathways in OSA using enrichment plots **(A)** and ridge plots **(B)**. **(C)** Gene set variation analysis (GSVA) for significantly enriched pathways in OSA.

To further explore the signaling pathway associated with immune genes involved in OSA, we identified the pathways significantly enriched through GSEA and GSVA analysis. GSEA analysis indicated that OSA is predominantly associated with an IL-17 signaling pathway, pertussis, rheumatoid arthritis, parathyroid hormone synthesis, secretion, and action, TNF signaling pathway, amyotrophic lateral sclerosis, and non-alcoholic fatty liver disease (Figures 6A,B). This result was consistent with the analysis of KEGG pathways that the IL-17 signaling pathway may play an important role in OSA. It is noteworthy that the non-alcoholic fatty liver disease signaling pathway is involved in the genesis of OSA disease.

The GSVA result is presented in the heat map (Figure 6C), and further uncovered differences of normal and OSA samples. Consistent with the result of the KEGG pathway, toll-like receptor signaling pathway, MAPK signaling pathway, and T cell receptor signaling pathway are associated with OSA.

### Prognostic Model Building and Validation

**FIGURE 7:**
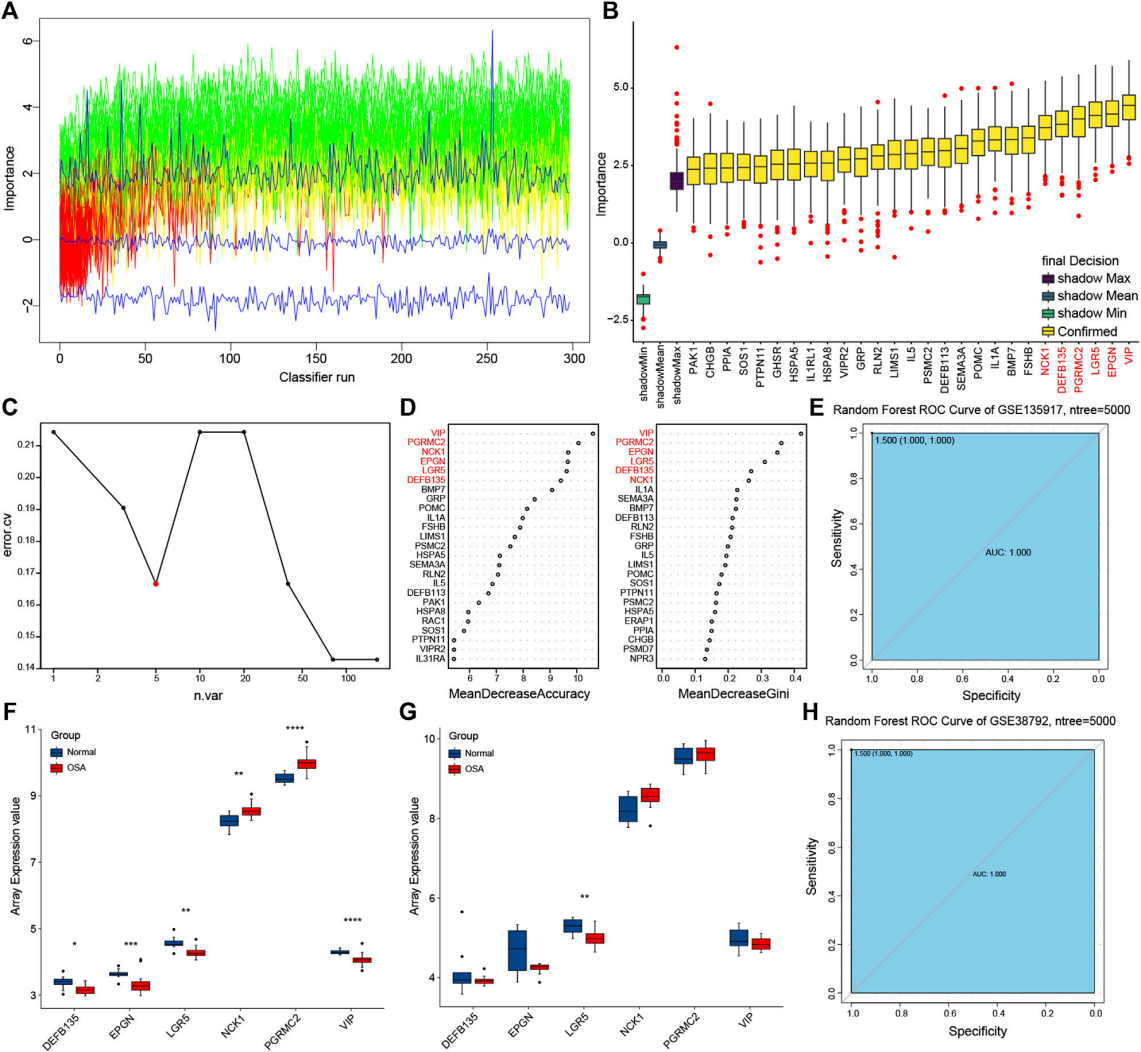
Prognostic model building and validation. **(A)** The z score evolution with Boruta run. **(B)** Selected genes by Boruta algorithm. **(C)** The average error rate of random forest model. **(D)** Variable importance ordered by accuracy and the gini index of a mean decrease in random forest. **(E)** Receiver operating characteristic (ROC) curve with area under the curve (AUC) values for GSE135917. **(F)** The expression of predictors in GSE135917. **(G)** The expression of predictors in GSE38792. **(H)** ROC curve with AUC values for GSE38792.

Boruta feature selection method was used to select significant predictors to improve prediction in immune-related DEGs. Along with Boruta running, the z score evolution is shown in Figure 7A. A total of 27 genes were selected by the Boruta algorithm (Figure 7B). Random forest analysis provided further support for the predictors’ selection (Figures 7C,D). The average error rate was minimum with five sample trees from Figure 7C. Then we analyzed the variable importance of random forest by using accuracy and the Gini index of a mean decrease (Figure 7D). Therefore, we built the final prognostic predictors with the top six genes, that were vasoactive intestinal peptide (*VIP*), progesterone receptor membrane component 2 (*PGRMC2*), NCK adaptor protein 1 (*NCK1*), leucine rich repeat containing G protein-coupled receptor 5 (*LGR5*), epithelial mitogen (*EPGN*), and defensin beta 135 (*DEFB135*). ROC curves were also applied to compare the efficiency of the predictive model and those genes.

Training and testing sets were used for each evaluation to confirm the performance and reliability of the prognostic model (Figures 6E–H). The expression trend of the predictive genes in the training set (GSE135917) (Figure 7F) was consistent with the testing set (GSE38792) (Figure 7G). The AUC of the ROC for this prognostic model was 1 in both of the two sets (Figures 7E,H), indicating that these predictors showed good performance in distinguishing persons who will easily lead to OSA.

### Immune Cell Infiltrate Analysis

**FIGURE 8:**
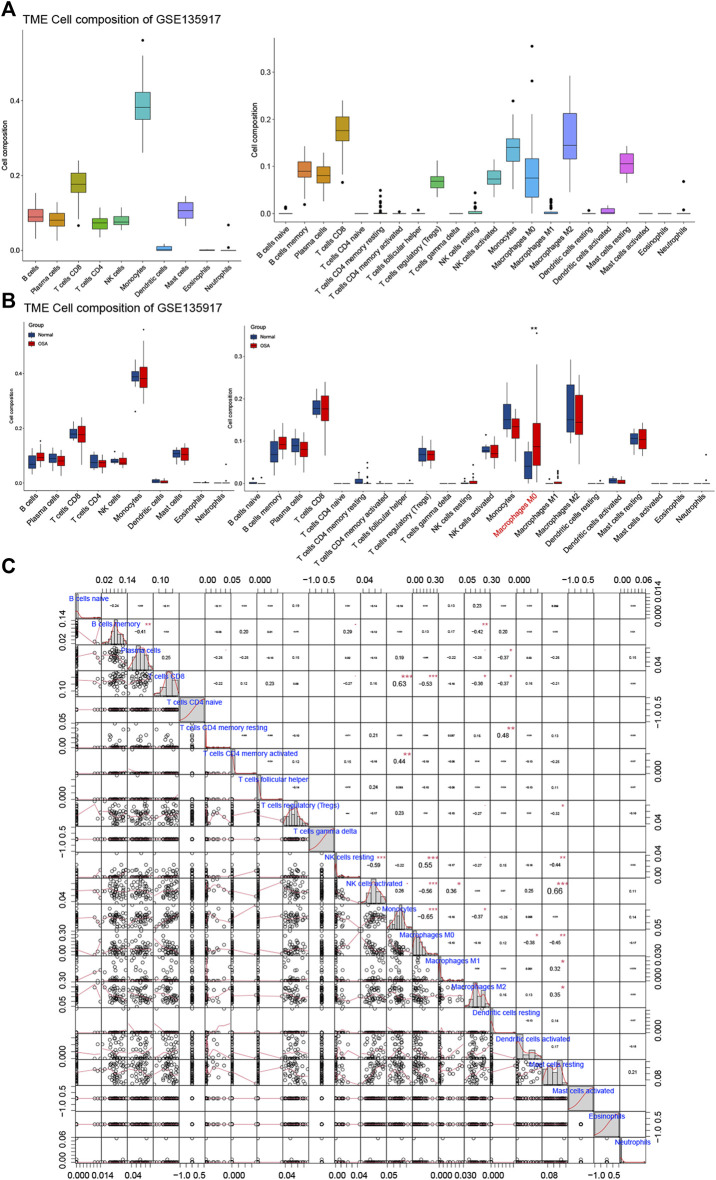
Immune cell infiltrate analysis. **(A)** The composition of the immune cell infiltrate in OSA. **(B)** The differential expression of different types of immune cells between normal and OSA tissues. **(C)** Correlation matrix of 22 types of immune cell proportions.

We investigated whether distinct patterns of immune infiltration could be discerned based on the 10 kinds and 22 types of the immune cell by the CIBERSORTx method. First, we evaluated the composition of the immune cell infiltrate in OSA (Figure 8A). In adipose tissue, the predominant immune cell type was monocytes, followed by CD8 T cells. The main cell types of monocytes kind were monocytes, anti-inflammatory macrophages (M2), and inactive macrophages (M0), with almost no inflammatory macrophages (M1).

The differential proportions of immune infiltration cells in normal and OSA groups are shown in Figure 8B. Within the 10 kinds of immune cells, there were no significant differences in immune cell composition between OSA patients and normal, whereas when divided into the 22 types, macrophages M0 was statistically significantly different between OSA patients and normal. Macrophages M0 was significantly higher in the adipose tissue of OSA patients compared with normal.

Furthermore, Pearson correlation analysis was used to investigate the correlations of immune cells in the training set (Figure 8C). We observed that monocytes had a significant positive correlation with CD8 T cells, and a significantly negative with macrophages M0, macrophages M2. Meanwhile, T cells CD8 was negatively correlated with macrophages M0 and macrophages m2. The relationship between CD8 T cells and monocytes, as well as the polarization of monocytes, requires further study. In addition, there was a positive correlation between NK cell activated and mast cell resting.

### Correlation Analysis Between Predictors and Immune Cells

**FIGURE 9:**
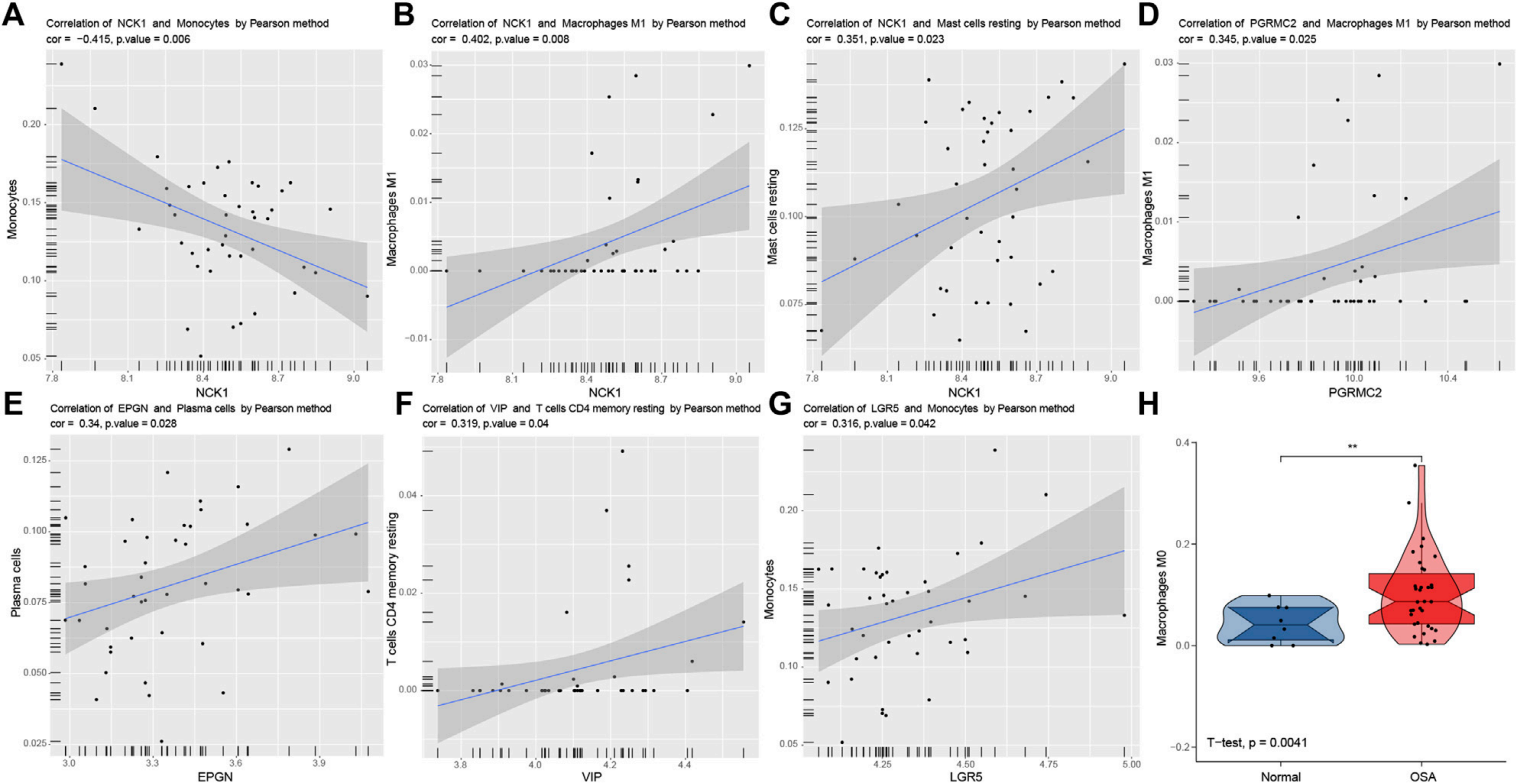
Correlation analysis between predictors and immune cells. Significantly correlations between predictors and immune cells: NCK1 and monocytes **(A)**, NCK1 and macrophages M1 **(B)**, NCK1 and mast cells resting **(C)**, PGRMC2 and macrophages M1 **(D)**, EPGN and plasma cells **(E)**, VIP and T cells CD4 memory resting **(F)**, and LGR5 and monocytes **(G,H)**. Violin plots of the abundance of macrophages M0. The box plots in the violin indicate the median and interquartile range of the data distribution.

Significant correlations between six predictive genes and immune cells are shown in Figures 9A–G. The abundance of macrophages M0 in normal and OSA groups is illustrated using violin plots (Figure 9H). *NCK1* had a significant correlation with more immune cells than the other five genes. There was a significant negative correlation between *NCK1* and monocytes, which was following the results in front. That indicated that *NCK1* may be associated with monocytes and subsequent polarization in the adipose tissue of OSA patients.

### Transcription Factors and Target Drugs Analysis

**FIGURE 10:**
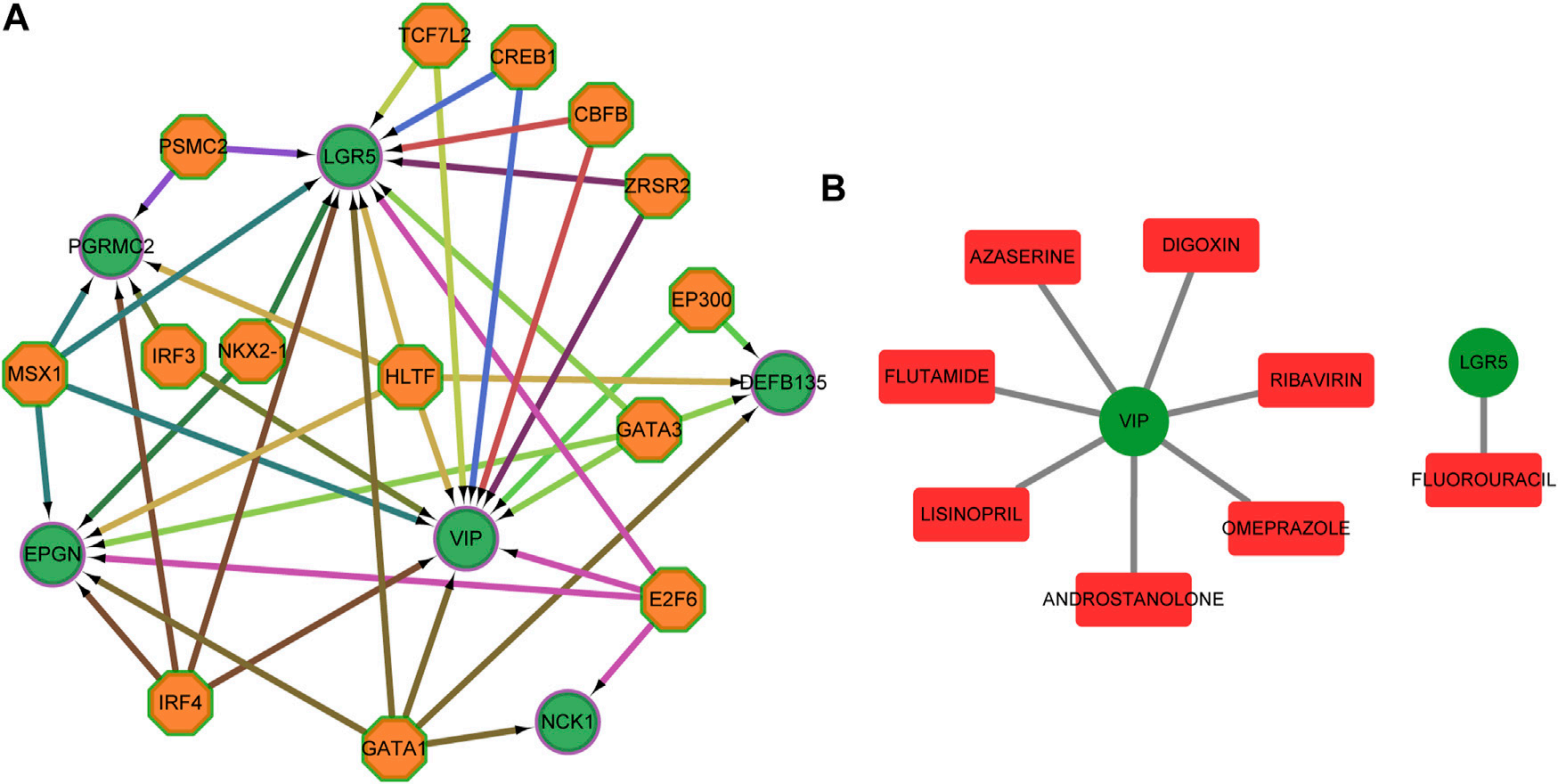
Transcription factors and target drugs analysis. **(A)** Regulatory network of the predicted transcription factors and the target genes. **(B)** Drug-gene network using gene-centric fashions. Green circles indicate target genes, orange octagons indicate predictive transcription factors, and red quadrilateral indicate predictive drug.

Candidate transcription factors, being hypothetically able to control the expression of the six predictors, were predicted (Figure 10A). We also used DGIdb to identify essential genes that are potentially “druggable”. Figure 10B shows a drug-gene network visualization using gene-centric fashions.

### Analysis of the Expression Level of the Predictive Genes *In Vivo*.

**FIGURE 11:**
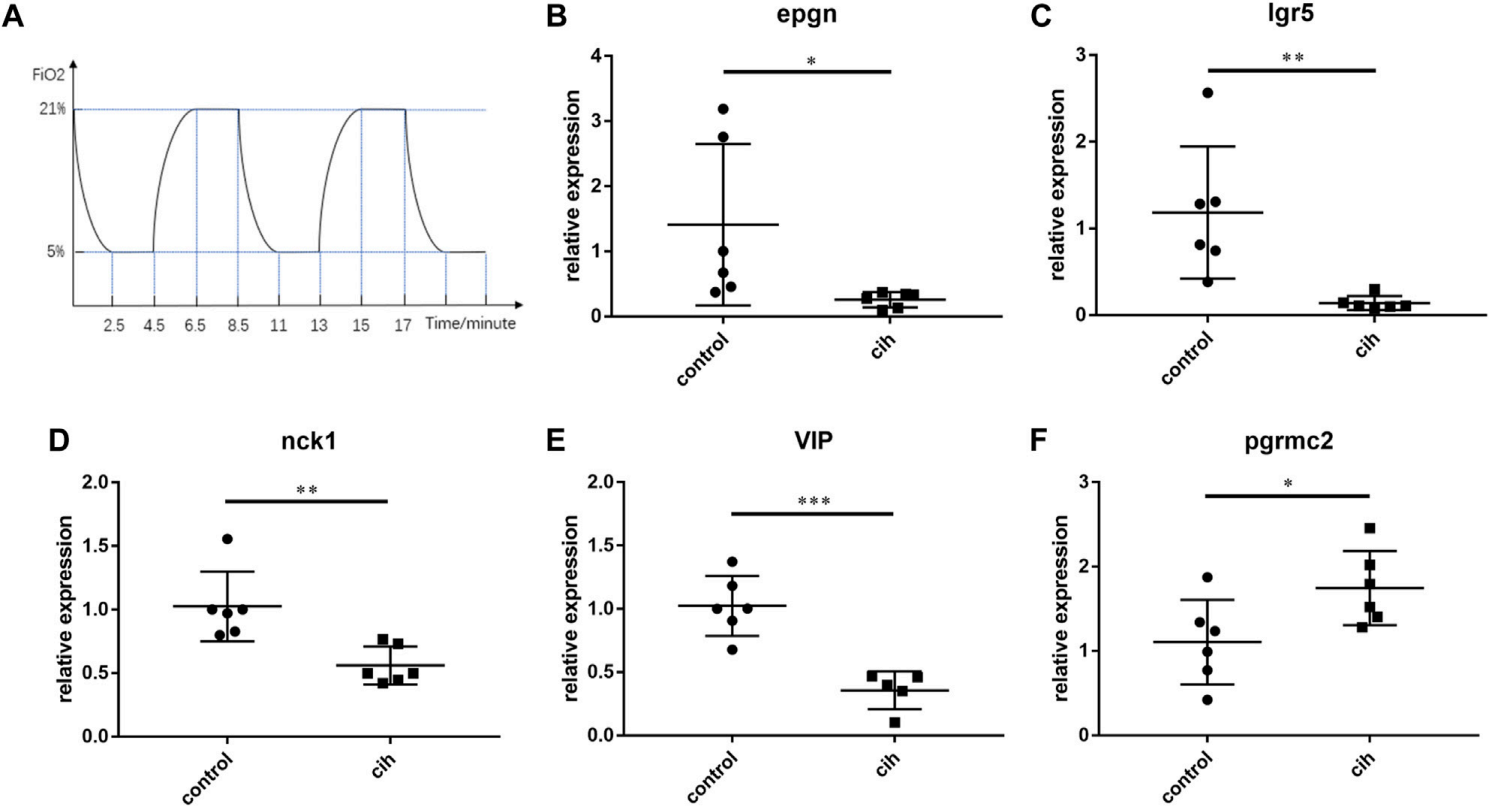
The expression trend of prognostic factors after 4 weeks of chronic intermittent hypoxia (CIH). **(A)** The flow chart of CIH exposure procedure. The expression of EPGN **(B)**, IGR5 **(C)**, NCK1 **(D)**, VIP **(E)**, and PGRMC2 **(F)**. Data are presented as means ± SEM, n = 6 for each group. *p < 0.05, * *p < 0.01 and * * *p < 0.001 compared with control animals.

Mice exposed to chronic intermittent hypoxia (CIH), which mimic hypoxia condition during OSA, are most frequently used as an animal model for OSA. The flow chart of CIH exposure procedure in our study was shown in Figure 11A. As *DEFB135* was not expressed in mice, the expression of the other five predictive genes after chronic intermittent hypoxia for 4 weeks is shown (Figures 11B–F). The levels of *EPGN*, *LGR5*, *NCK1,* and *VIP* in visceral adipose tissue of CIH group mice were significantly down-regulated compared to the control group while the level of *PGRMC2* was significantly up-regulated.

## Discussion

Obesity is a clear risk factor in the development of OSA, the incidence rate of OSA is increasing year by year with the prevalence of obesity. The synergistic effect of obesity and OSA increased the incidence of metabolic diseases, such as dyslipidemia, hypertension, insulin resistance, cardiovascular diseases, and non-alcoholic fatty liver disease. (Jordan et al., 2014). Recently, OSA is considered to be an independent factor affecting lipid metabolism and there has been a great interest in the interaction between OSA and lipid metabolic dysfunction. Rodents are used to study the occurrence and development mechanism of dyslipidemia in OSA but they do not naturally exhibit OSA. Animal models to research the physiological mechanisms underlying OSA always achieve its hallmarks outcomes as intrathoracic pressure swings, sleep fragmentation, hypercapnia, and intermittent hypoxia (Barros and García-Río, 2019; Mesarwi et al., 2019).

Inflammation and lipid signaling are synergism to maintain the stability of the internal environment and immunity (Shimobayashi et al., 2018), so the dyslipidemia of OSA may be the result of the immune response. Therefore, we performed an integrated analysis to identify the effect of immune-related genes on dyslipidemia in OSA. In the first part of our study, the results of the PCA analysis showed that normal and OSA adipose tissue samples could be clearly distinguished by the immune-related DEGs as well as DEGs. It laterally proved that immune responses contribute to the pathogenesis and progress in adipose tissue of OSA.

Next, we used the immune-related DEGs to build a PPI network and find the functional similarity between them. GO, KEGG, DO, GSEA, and GSVA analyses were performed to explore the biological functions, enriched signaling pathways, and related diseases. IL-6, the highest ranked gene in the PPI network, was significantly downregulated in cytokine-cytokine receptor interaction and lipid and atherosclerosis signaling pathways. MAPK3, one of top3 hub genes in PPI network, is a member of the MAP kinase family. MAP kinases, also known as extracellular signal-regulated kinases (ERKs), act in a signaling cascade that regulates various cellular processes such as proliferation, differentiation, and cell cycle progression in response to a variety of extracellular signals. Protein kinase C and ERK activation are required for TFF-peptide-stimulated bronchial epithelial cell migration and tumor necrosis factor-alpha-induced interleukin-6 (IL-6) and IL-8 secretion (Graness et al., 2002). The IL6-202 and IL6-205 transcripts that confer drug resistance to Vemurafenib by reactivating the MAPK pathway while IL6-201 is not responsible for the resistance in A375 melanoma cells. Neutralizing IL-6 significantly increased the sensitivity of drug-resistant cells to Vemurafenib (Zhao et al., 2020). From our results, those two pathways were the top3 pathways negatively correlated with the OSA group. It is the same as a previous meta-analysis that either children or adults with obstructive sleep apnea syndrome had higher serum/plasma IL-6 levels compared to healthy controls (Imani et al., 2020). Results of GO BP terms, KEGG pathway, and GSVA revealed that immune-related DEGs were mainly enriched in cell adhesion and T cell-mediated immunity pathways, according to well with the pathophysiological mechanism of DO term enriched diseases. Notably, the IL-17 signaling pathway, the result enriched both in KEGG analysis and GSEA, is IL-17 family mediated immune response in both acute and chronic inflammatory responses. Clinical evidence indicated that the pathogenesis of OSAS may be associated with increased IL-17A broken the balance of peripheral Th17/Treg (Ye et al., 2012; Ying et al., 2014). Following clinical observation (Jordan et al., 2014; Chung et al., 2021; Yeghiazarians et al., 2021), disease ontology (DO) queries revealed multiple DO terms not only associated with inflammation, but also with metabolic diseases such as cardiovascular diseases and liver diseases. GSEA analysis showed the immune-related DEGs significantly enriched in pathways related to non-alcoholic fatty liver disease, maybe it was the combined action of obesity and OSA in lipid metabolism.

In the following part of our study, we selected significant diagnostic genes (*VIP, PGRMC2, NCK1, LGR5, EPGN, and DEFB135*) and constructed a diagnostic model using the genes. Concerning diagnostic value, the AUC of the diagnostic model and the expression of the six diagnostic genes were analyzed using cross-validation. The results showed that those six genes may be promising targets for the diagnosis of dyslipidemia of OSA. Although none of the genes has been reported to be associated with OSA, it is also reasonable for them to influence the development of dyslipidemia in OSA. *PGRMC2*, ubiquitous expression in fat, is involved in adipose tissue development and steroid hormone mediated signaling pathway (Galmozzi et al., 2019). As metabolic molecules, it is not surprising that they could be involved in the lipid metabolism of OSA. Vasoactive intestinal peptide (*VIP*) has immune regulatory functions, and administration of *VIP* can inhibit experimental colitis (Sun et al., 2019). *EPGN* is a member of the epidermal growth factor family, and EPGN SNP (single nucleotide polymorphism) is significantly associated with variations in cytokine secretion to vaccinia virus stimulation in smallpox vaccine recipients (Kennedy et al., 2012). *LGR5*, a receptor for R-spondins, promotes epithelial-mesenchymal transition by activating the Wnt/β-catenin pathway in glioma (Zhang et al., 2018). *NCK1* is involved in enhancing downstream T cell activation signaling (Wipa et al., 2020). *DEFB135* also was one of the diagnostic genes, while *DEFB129* got the highest scores in functional similarity analysis. Both of those two genes are a member of the beta defensin protein family, and defensins are the only group of antimicrobial peptides found in animals, involved in the first line of defense in their innate immune response against pathogens (Xu and Lu, 2020). By now, the researches of beta-defensins mainly focus on infection (Xu and Lu, 2020) and reproductive (Batra et al., 2019), and there is no report on the relationship between beta-defensins and lipid metabolism. Although not directly be regulated in lipid metabolism, the latter four diagnostic genes have been reported to be involved in inflammation. Infection and inflammation are associated with marked changes in lipid and lipoprotein metabolism (Khovidhunkit et al., 2004). Those changes may through effecting on liver lipid synthesis, adipose tissue lipolysis, and postprandial lipid clearance lead to dyslipidemia in OSA (Barros and García-Río, 2019).

To further explore the reason for the changes of immune molecules, we analyzed the immune microenvironment of adipose tissue in the OSA group and then seek out the relationship of diagnostic immune genes and immune cells. In our research, monocytes were mainly enriched kinds of immune cells. Among the kind of monocytes, the proportion of macrophages M0 in the OSA group was significantly higher than that in the normal group, while the ratios of macrophages M1 were almost no expression. This suggested that the major immune cell involved in dyslipidemia of OSA is the macrophage. Macrophages, belonging to the monocyte-macrophage system, modulate inflammatory responses and microbial killing. Macrophages need to display function plasticity to respond to different microenvironmental. Inflammatory stimuli such as lipopolysaccharide (LPS) and interferon-γ (IFN-γ) induce classically activated (M1) macrophages, and anti-inflammatory cytokines such as interleukin-4 (IL-4) or IL-13 induce an alternatively activated (M2) macrophages (Motwani and Gilroy, 2015; Saha et al., 2017). The lipid metabolism signaling pathway and its products play a key role in regulating macrophage polarization (Saha et al., 2017). In *in vitro* experiment, M2 macrophages depend on fatty acid oxidation whereas M1 macrophages depend on an increase in glycolysis (Tabas and Bornfeldt, 2016; Saha et al., 2017; Shimobayashi et al., 2018). The relationship between macrophage phenotypic states and pathological conditions of metabolism disease have been demonstrated in numerous studies. Macrophages play important roles in all stages of atherosclerosis, and pure M1 and M2 macrophages almost certainly do not occur in atherosclerotic lesions. Early in the disease, macrophages accumulate in susceptible regions of arteries. When macrophages are exposed to a plethora of stimuli, they differentiate into different types and play different roles (Tabas and Bornfeldt, 2016). M1 macrophages dominated the rupture-prone shoulder regions of the plaque while increasing M2 activation was displayed in vascular adventitial tissue (Motwani and Gilroy, 2015). Obesity is considered chronic tissue information and causes insulin resistance. In obesity, the balance is tilted toward the M1-like macrophage polarization state (Shimobayashi et al., 2018). In our study, extremely no expression of macrophages M1 further confirmed that dyslipidemia in OSA is not simply caused by obesity. We found that the cells of the monocyte-macrophage system were mainly composed of monocytes, macrophages M0, and macrophages M0 in adipose tissue of OSA. Only macrophages M0 was significantly different between normal and OSA group, and it can onset of polarization adopting variable states of activation.

In adipose tissue, the second immune cell type was CD8 T cells. CD8 T cells are key members of adaptive immunity and immunological memory. The control of lipid metabolism is central to the appropriate differentiation and functions of T lymphocytes (Howie et al., 2017), and it was according to our result that T cell pathways mainly enriched in adipose tissue of OSA. In early atherosclerosis, CD8 T cells control monopoiesis and macrophage accumulation and contribute to macrophage cell death in atherosclerotic plaques (Schäfer and Zernecke, 2020). While in our study, CD8 T cells were positively correlated with Macrophages M0, Macrophages M2, and negatively correlated with monocytes. Regulatory pathways between the macrophage subsets and other immune cells need to be further studied to help us better understand the mechanism of dyslipidemia in OSA.

In the animal model experiment, the expression trend of *LGR5, EPGN, PGRMC2,* and *VIP* were consistent with the conclusion of the testing set (GSE38792) and the training set (GSE135917). Notably, *NCK1* had the opposite trend with the conclusion of the two sets. Perhaps *NCK1* is a good molecule of penetration. As a prognostic gene associated with various immune cells, *NCK1* is highly expressed in adipose tissue. *NCK1* covers aspects of tissue development and homeostasis, invasiveness of tumor cells, and immune cell function. When T-cell antigen receptor (TCR) is triggered, *NCK1* is recruited to the CD3ε subunit of the TCR then switches on downstream T-cell activation pathway (Wipa et al., 2020). In our study, *NCK1* was significantly negatively correlated with monocytes and positively correlated with macrophages M1. Perhaps because the activated T cell response of *NCK1* initiates activated inflammatory macrophages (M1), leading to the corresponding decrease of monocytes.

Moreover, we analyzed candidate transcription factors and target drugs for the prognostic genes. We discovered that *LGR5* might be the likely target gene for the treatment of dyslipidemia in OSA. *LGR5* was the only predictive gene with a significant difference in our study and the two datasets. It also showed a positive correlation with monocytes. Further analyses were necessary to analyze the role of *LGR5* in the polarization of macrophages in the lipid metabolism of OSA.

The current work is the first to investigate the role of immune-related genes in the pathogenesis of dyslipidemia in OSA patients through bioinformatics methods. However, there were still several limitations to our research. First, to clarify the role of lipid metabolism in OSA, all kinds of clinical factors should be considered, such as whether obesity, the severity of intermittent hypoxia, daily physical activity, and fasting-fed state. Second, we have not verified the expression of key immune genes in clinical samples. At the last, the sample size of our data was relatively small, which may affect the gene expression in OSA. More clinical characteristics of OSA are needed to be included in the study for further analysis. The public datasets in our study had small sample size of control group and there was gender bisa. Therefore, we aim to use more samples and perform further experiments to confirm the potential mechanism and clinical utility. First, it would be interesting to examine basic expression of these predicted genes with western blot, IHC, IF assays and so on. Second, to clarify the function of the genes in OSA, a clean loss-of-function and gain-on-function study with tissue-type specificity and cell-type specificity remains warranted. A recent series of molecular experiments may prove strong evidence for the possible phenotype and pathway regulation of these predicted genes. Third, the co-expression and interaction among predicted genes is a new exciting Frontier that awaits further investigation.

## Conclusion

The clinical evidence confirms the link between OSA and dyslipidemia. However, because of a bias for clinicians who may not consider routine screening for OSA in lean individuals, some dyslipidemia caused by OSA cannot be identified early. Our study demonstrated strong associations between immune genes and the development of dyslipidemia in OSA. Six prognostic genes were found and showed great testing efficacy. The analysis between immune filtration landscape and prognostic genes revealed those genes may affect the polarization of macrophage and differentiation of T lymphocyte subsets brought about abnormal lipid metabolism in OSA. This work will contribute to explore the relationship between inflammation and dyslipidemia, thus promoting our understanding of the molecular mechanisms of lipid metabolism in OSA and may offer potential targets for the regulation of lipid metabolism and treatment of adipose dysfunction. Additionally, the expression of *EPGN*, *LGR5*, *NCK1, PGRMC2*, and *VIP* were also comfired at transcriptional using qPCR, indicating that those genes are closely linked to dyslipidemia in OSA. However, the limitations of our study are lacking in the function validation of the genes such as loss-of-function and gain-on-function study. Therefore, further functional investigations of these targets are essential. The roles of the prognostic genes in different macrophage subsets and other immune cell metabolic properties require further experimental validation.

## Data Availability

The datasets presented in this study can be found in online repositories. The names of the repository/repositories and accession number(s) can be found below: http://www.ncbi.nlm.nih.gov/geo/GSE135917 and GSE38792
